# Body composition in Nepalese children using isotope dilution: the production of ethnic-specific calibration equations and an exploration of methodological issues

**DOI:** 10.7717/peerj.785

**Published:** 2015-03-03

**Authors:** Delan Devakumar, Carlos S. Grijalva-Eternod, Sebastian Roberts, Shiva Shankar Chaube, Naomi M. Saville, Dharma S. Manandhar, Anthony Costello, David Osrin, Jonathan C.K. Wells

**Affiliations:** 1 Institute for Global Health, University College London, London, UK; 2 Mother and Infant Research Activities, Kathmandu, Nepal; 3 Childhood Nutrition Research Centre, Institute of Child Health, University College London, London, UK

**Keywords:** Anthropometry, Nepal, Child, Body composition, Bioelectrical impedance

## Abstract

**Background.** Body composition is important as a marker of both current and future health. Bioelectrical impedance (BIA) is a simple and accurate method for estimating body composition, but requires population-specific calibration equations.

**Objectives.** (1) To generate population specific calibration equations to predict lean mass (LM) from BIA in Nepalese children aged 7–9 years. (2) To explore methodological changes that may extend the range and improve accuracy.

**Methods.** BIA measurements were obtained from 102 Nepalese children (52 girls) using the Tanita BC-418. Isotope dilution with deuterium oxide was used to measure total body water and to estimate LM. Prediction equations for estimating LM from BIA data were developed using linear regression, and estimates were compared with those obtained from the Tanita system. We assessed the effects of flexing the arms of children to extend the range of coverage towards lower weights. We also estimated potential error if the number of children included in the study was reduced.

**Findings.** Prediction equations were generated, incorporating height, impedance index, weight and sex as predictors (*R*^2^ 93%). The Tanita system tended to under-estimate LM, with a mean error of 2.2%, but extending up to 25.8%. Flexing the arms to 90° increased the lower weight range, but produced a small error that was not significant when applied to children <16 kg (*p* 0.42). Reducing the number of children increased the error at the tails of the weight distribution.

**Conclusions.** Population-specific isotope calibration of BIA for Nepalese children has high accuracy. Arm position is important and can be used to extend the range of low weight covered. Smaller samples reduce resource requirements, but leads to large errors at the tails of the weight distribution.

## Introduction

Malnutrition in childhood is highly prevalent worldwide. In 2011, global estimates showed 165 million children were stunted and 43 million were overweight (Black et al., 2013; WHO, 2009). The short- and long-term consequences of malnutrition, such as increased risk of infection, reduced survival, impaired development, and increased risk of chronic disease, make accurate assessment of nutritional status crucial (Black et al., 2008; Victora et al., 2008). Body composition analysis in childhood is one such method, as it is both a marker of current health (Wells & Fewtrell, 2006) and a predictor of disease later in life (WHO, 2009). High-precision methods for assessing body composition such as water or air displacement are currently deployed in clinics and research institutions. However, most of these methods are unsuitable for large-scale epidemiological studies as they are either impractical or expensive (Snijder et al., 2006).

Bioelectrical impedance analysis (BIA) is a relatively simple, inexpensive, and accurate method suitable for large epidemiological studies of body composition in healthy subjects, and for assessing fluid distribution and changes in both healthy and unhealthy subjects (De Lorenzo & Andreoli, 2003). BIA requires valid equations obtained from calibration-studies to derive lean mass (LM) from electrical impedance. However, most equations built into BIA instruments were validated in European or American populations, raising the question of validity in other populations (Haroun et al., 2010).

We set out to calibrate the Tanita BC-418 using deuterium oxide for children aged 7–9 years in Nepal. In an attempt to improve accuracy and inform similar future studies, we also explored the effects that procedural changes would have on the results.

## Methods

### Study sample

The calibration study formed a component of a larger follow-up study of 841 children (406 girls) aged 7–9 years from a cohort born to mothers who received antenatal micronutrient supplements during pregnancy, in Dhanusha district, Nepal (Osrin et al., 2005). We recruited children for the calibration study from the birth cohort, and other children from three local schools and neighbourhoods in Janakpur, the district capital. We used the parameters from the first 200 children already followed-up in the birth-cohort study to define the requirements of the calibration study: weight range 14–34 kg and age 7–9 years. In addition, all children were at a pre-pubertal stage of development. To optimise the accuracy of the resulting prediction equation, we aimed to purposively sample a total of 50 boys and 50 girls, equally distributed in 2 kg weight bands.

#### Anthropometry

To limit inter-observer variation, measurements were obtained by two trained data collectors. Height was measured with a Leicester stadiometer, accurate to 0.1 cm and calibrated with a 50 cm calibration rod. The child was positioned with feet together, heels touching the stadiometer, knees extended and head in the Frankfort plane. Weight was measured with the Tanita BC-418 (Tanita Corporation, Tokyo, Japan), accurate to 0.1 kg and calibrated with standard weights. Children wore light underwear and were given a vest and sarong that together weighed 200 g. Measurements were made after emptying the bladder.

#### Bioelectrical impedance analysis

BIA was performed using the Tanita BC-418. It has 8 electrodes: two on each hand and foot plate, which pass an imperceptible alternating current (frequency 50–60 Hz) from one to another, measuring the impedance. A 200 g weight adjustment for clothing was applied. Measurements were conducted at a similar time of the day, but it was not possible to conduct fasting measurements due to the logistic difficulties of getting children to the office.

BIA readings were not available from some small children because they appeared to have impedance values above the 1,200 Ohm cut-off chosen for European populations, despite the equipment’s reported age range of 7–99 years (Tanita Corp, 2011). As flexing the arms to 90° lowered the impedance and enabled us to obtain measurements from smaller children, we tested whether this method would produce adequate results with an acceptable level of error. Two BIA recordings were obtained, one with elbows flexed at 90° and one with elbows extended at 180° (hereafter 90° or 180° arm positions), with the child standing in the anatomical position.

#### Isotope administration

All children were invited to our facilities in the morning and time of arrival was recorded. Each child was monitored by a member of staff until they left the building. The first of two saliva samples was taken at least half an hour after entry. The child was advised to collect some saliva in their mouth and was given a salivette to roll in their mouth for approximately two minutes. They were asked not to chew the bud and to make it as wet as possible. When the salivette appeared wet enough, the child replaced it in the container and it was centrifuged immediately at 3000 rpm for at least three minutes. The process was repeated if more saliva was needed. The saliva was then pipetted into pre-labelled 2 ml microtube bottles.

The child was then given a pre-prepared and pre-labelled drink of bottled water containing approximately 125 ml of water with 1.2 ml (0.06 mg/kg, assuming an average weight of 20 kg) of 99.8% sterility tested deuterium oxide (CK Gas Products Ltd, Leicestershire, UK). Isotope dilution with deuterium is widely used in medical research in all age groups. Deuterium is a non-toxic, non-radioactive, stable isotope of water without any known associated risk in humans at this concentration (International Atomic Energy Agency, 2009). The bottle and straw used to administer the isotope dose were weighed with the plastic bag containing them before and after, using an Ohaus Traveler TA152 weighing balance (Ohaus Corp, Parsippany, New Jersey, USA) accurate to 0.01 g.

The children were then taken into a separate room where they were given biscuits and a standard drink (230 ml). Additional food or drink intake was not permitted in the period following dose administration until a second saliva sample was obtained, in a similar way to the first, four hours later. Saliva samples were stored in a freezer and transported frozen to the UK.

#### Total body water estimation analysis

The samples were analysed by Iso-Analytical (Iso-Analytical Ltd., Crewe, UK). Total body water (TBW) was estimated using continuous flow isotope ratio mass spectrometry (Thermo Fisher Gasbench-Delta XP system; Thermo Scientific, Waltham, Massachusetts, USA). Samples were tested in duplicate using the equilibration technique, as follows: a sample was pipetted into Exetainer tubes containing 5% platinum on alumina, the tubes were sealed, filled with pure hydrogen and left to equilibrate for at least eight hours, after which hydrogen enrichment would be proportional to the water. Samples were measured against three reference standards prepared in the same way.

### Analysis

(i) *Prediction equations.* TBW and LM were calculated using Eqations A and B in Text S1. Fat mass was calculated as the difference between LM and body weight (kg) and the body mass index (BMI) as body weight (kg) divided by height (m) squared. The calibration prediction equations were generated using stepwise multivariable linear regression, models with LM as the dependent variable. While impedance index predicts LM, the coefficient of determination (*R*^2^) can be improved by adding other variables. Predictor variables were added to the model with assessment of goodness of fit.
(ii) *Comparison of our estimates with the Tanita system.* Agreement of our estimates and those provided by the Tanita system’s built-in equations was assessed using the Bland–Altman method (Bland & Altman, 1986).
(iii) *Assessment of the effect of changing arm position.* Separate prediction equations were generated for 90° and 180° arm positions. We assessed the difference in estimates between these two equations when applied to the whole cohort; and also when limited to children <16 kg, in whom the 90° arm position may be useful.
(iv) *Post-hoc analysis: assessment of the effect of reducing the range of weights sampled.* We aimed to find children spanning all weight categories observed in our larger cohort in order to optimise regression lines across the range of body weights. This approach adds to the time and cost required for conducting an isotope calibration study. Therefore, we looked at whether reducing the number of children included in the study makes a difference to the prediction equations generated in order to understand how much of a reduction in the sample would be tolerable in practice. We examined this in two ways. First, by removing children from the total sample in order to artificially reduce the range of weights covered to 1.5 (*n* = 89), 1.25 (*n* = 76), 1.0 (*n* = 66), and 0.75 (*n* = 50) standard deviations from the mean weight. Second, by artificially reducing the concentration of children at the tails of the body weight range. For this we randomly removed half the children whose weights were >1SD or <-1SD from the mean. The process was repeated four times to produce four datasets with 83 data points each. Prediction equations were generated for each new dataset and estimates of lean mass were obtained from our larger cohort of children as described in the Study sample section above. Agreement between estimates was evaluated using Bland–Altman plots.

Statistical analysis was carried out in Excel (Microsoft Corp, Redmond, Washington, USA), Prism (GraphPad Software Inc, La Jolla, California, USA) and Stata (StataCorp, College Station, USA) software.

### Ethics statement

The project was approved by the University College London research ethics committee (Project ID number 2744/001) and the Nepal Health Research Council (Reference 51/2011). We showed participants and their guardians a film about the calibration study and the reasons for doing it, produced on site. The parents or guardians of all participants then gave written informed consent in their native language.

## Results

### Sample characteristics

We recruited 109 children. The procedures were not completed in two: one child had learning difficulties and was unable to provide a saliva sample; the other was found to be 10 years old after entering the study. From the remaining 107 children, samples from five were removed from mass spectrometry analysis as we had others in their weight categories, leaving a final sample of 102 children. We were unable to obtain BIA readings in the 180° arm position in two children. Both were light and their whole body impedance with arms at 90° was close to the Tanita BC-418’s maximum cut-off of 1200 Ohms. Table 1 shows characteristics of the sample. On average, boys were heavier than girls, (difference in mean weight 2.2 kg; 95% CI: 0.3, 4.2), but had similar height and BMI. The intra-observer technical error of measurement was 0.05% for both observers and the inter-observer coefficient of reliability was 0.97 for height.

**Table 1:** Characteristics of the sample. Population-specific isotope calibration of the Tanita BC-418 bioelectrical impedance machine for prediction of lean mass in Nepalese children.

	Boys	Girls
Number	50	52
Age (years)	8.7 (0.6)	8.6 (0.6)
Weight (kg)	23.3 (5.4)	21.0 (4.7)
Height (cm)	124.2 (9.4)	121.3 (8.9)
Trunk height (cm)	111.8 (4.4)	110.1 (4.4)
BMI (kg/m^2^)	14.9 (1.9)	14.4 (2.1)
TBW (L)	13.9 (2.8)	12.5 (2.5)
Lean mass (kg)	18.4 (3.7)	16.7 (3.3)
Fat mass (kg)	4.9 (2.5)	4.3 (1.9)
Fat mass %	20.4 (6.5)	20.1 (6.4)
Straight arms Z (Ohms)	903.3 (96.5)	990.0 (100.8)
Straight arms impedance index (cm^2^/Z)	17.5 (3.7)	15.3 (3.2)
Bent arms Z (Ohms)	844.1 (91.3)	934.7 (104.6)
Bent arms impedance index (cm^2^/Z)	18.7 (3.9)	16.1 (3.6)

*(i) Prediction equations*

The prediction equations generated for TBW and LM (with height in cm and impedance in Ohms) with a 180° arm position were:

(1) \begin{eqnarray*} \mathrm{TBW}=0.7146+1.5959~({\mathrm{height}}^{2}/\mathrm{impedance}) \end{eqnarray*}

Root mean squared error (RMSE) = 0.781, *R*^2^ = 0.92

(2) \begin{eqnarray*} \mathrm{LM}=2.2022+0.9406~({\mathrm{height}}^{2}/\mathrm{impedance}) \end{eqnarray*}

RMSE = 1.053, *R*^2^ = 0.91.

We added sex (coded 0 for female, 1 for male) and weight (kg) to generate the final prediction equation, Eq. (3). Previous research has shown these to be the best predictors to add to the model (height and BMI tend to be collinear with weight) (Wickramasinghe et al., 2008) *R*^2^ and RMSE results are shown in Table 2. Adding in weight and sex increased *R*^2^ from 0.91 to 0.93 and reduced RMSE from 1.053 to 0.949.

(3) \begin{eqnarray*} \displaystyle \mathrm{LM}&=&\displaystyle 1.9461+(0.6808\times {\mathrm{height}}^{2}/\mathrm{impedance})+(0.2105\times \mathrm{weight})\nonumber\\ \displaystyle &&\displaystyle +\, (-0.3629\times \mathrm{sex}) \end{eqnarray*}

RMSE = 0.949, *R*^2^ = 0.93.

**Table 2:** Population-specific isotope calibration of the Tanita BC-418 bioelectrical impedance machine for prediction of lean mass in Nepalese children. Alternative models to predict lean mass.

	Arms 180°		Arms 90°	
Predictors	*R* ^2^	Root mean squared error	*R* ^2^	Root mean squared error
Impedance index	0.91	1.053	0.92	1.044
Impedance index, sex of child	0.92	1.025	0.92	1.014
Impedance index, weight	0.93	0.960	0.93	0.954
Impedance index, sex of child, weight	0.93	0.949	0.93	0.942

(ii) *Comparison with the Tanita system estimates*

**Figure 1:**
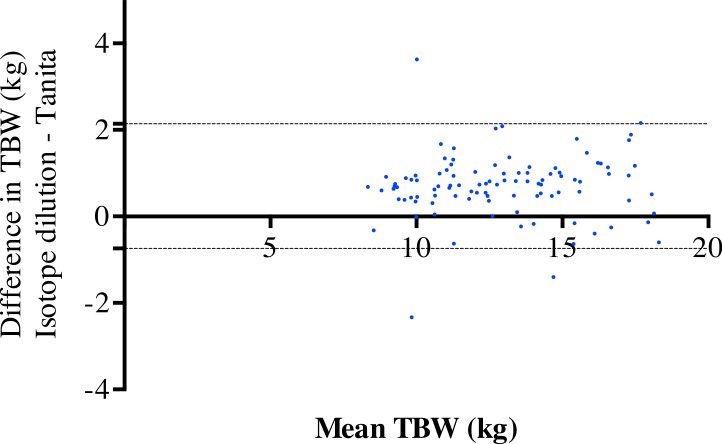
Comparison of isotope dilution and Tanita system. Bland–Altman plots showing the level of agreement for isotope dilution and the Tanita system methods for total body water.

Figure 1 shows the level of agreement for estimated TBW obtained using the equipment’s built-in equations and deuterium dilution. The mean bias was 0.70 kg and the 95% limits of agreement were −0.74 kg to 1.47 kg for TBW. The correlation was 0.03, indicating no change with increasing TBW. This resulted in a mean error of 385 g (SD 1,018 g) in LM, corresponding with a 2.2% error. The largest error was 4.54 kg (25.8%).

*(iii) Assessment of the effect of changing arm position*

Prediction equations were generated for TBW and LM using the 90° arm position:

(4) \begin{eqnarray*} \mathrm{TBW}~(\mathrm{arms}~90\deg )=0.6667+1.6324~({\mathrm{height}}^{2}/\mathrm{impedance}) \end{eqnarray*}

RMSE = 0.775, *R*^2^ = 0.92

(5) \begin{eqnarray*} L M~(\mathrm{arms}~90\deg )=2.2535+0.8774~({\mathrm{height}}^{2}/\mathrm{impedance}) \end{eqnarray*}

RMSE = 1.044, *R*^2^ = 0.92

(6) \begin{eqnarray*} \displaystyle \mathrm{LM}~(\mathrm{arms}~90\deg )&=&\displaystyle 1.9803+(0.6383\times {\mathrm{height}}^{2}/\mathrm{impedance})+(0.2084\times \mathrm{weight})\nonumber\\ \displaystyle &&\displaystyle +(-0.3773\times \mathrm{sex}) \end{eqnarray*}

RMSE = 0.942, *R*^2^ = 0.93.

Equations (3) and (6) are shown in Fig. S1 as scatterplots of impedance index against LM for the 180° and 90° arm positions. The graphs show the line of best fit with 95% confidence intervals (showing where the true line would be 95% of the time) and 95% prediction lines (showing where future data points are likely to be 95% of the time).

The mean impedance values with arms at 90° were lower in girls (63.9 Ohms; 95% CI: 58.8, 68.9) and boys (59.2 Ohms; 95% CI: 54.0, 64.4) than with arms at 180°. Using Eqs. (3) and (6) to estimate LM in the larger cohort (mentioned above in the study sample section) gave mean values of 17.32 kg (SD: 2.5 kg) and 17.25 kg (SD: 2.4 kg), respectively, with a difference of 70 g (95% CI: 55, 86 g; *p* < 0.01). The largest difference observed was 321 g, equivalent to a 1.9% error in LM. When limiting the comparison to children weighing <16 kg (*n* = 32), the mean difference was 19 g (95% CI: −29, 67 g; paired *t* test *p* 0.42).

If BIA values are obtained with arms at 90°, but estimates obtained using the standard 180° equation, the resulting error in LM is greater. The mean error rises to 694 g (95% CI: 676, 711 g), the largest difference being 1.55 kg (8.9% error). This is illustrated in the first graph in Fig. S2 in which the error increases with increasing LM values. When using a specific calibration for each arm position, the error reduces and is consistent across LM values,

*(iv) Reduction of the range of weights sampled.*

The study took two weeks to complete, but over half the children were recruited in the first two days. We looked at whether reducing the number of children included in the study made a difference to the prediction equations. In the first scenario we limited the data range collected, from all the data (*n* = 100) to 0.75 SD from the mean (*n* = 50), see Table S1. The prediction equations were similar until the data were limited to <1 SD. The error in LM increased from 0.7% to 1.3% as the numbers fell. Bland–Altman plots for all the data compared to the reduced weight range are shown in Fig. S3. Even with a small reduction to 1.5 SD from the mean (*n* = 89) at greater mean LM, the error increases. This effect is accentuated as the weight range narrows to 1.25 SD (*n* = 76), 1 SD (*n* = 66) and 0.75 SD (*n* = 50). No appreciable effect is seen at lower LM values until the sample reaches 0.75 SD.

In the second scenario, the central data remained, but data at the tails were reduced to half the data points >1SD from the mean (*n* = 83) (Table S2 and Fig. S4). This produced an error effect on LM of 0.1 to 0.7%. The Bland–Altman plots in Fig. S4 show a similar minimal error at low LM values, but large error at higher mean LM.

## Discussion

This study is among few that examine childhood body composition in low-resource settings, and we believe it is the first to generate prediction equations for BIA for a Nepalese population. BIA is an effective way of measuring body composition and is especially useful for large epidemiological projects, but population-specific calibration studies are needed as body composition is known to vary in different ethnic groups (Khan et al., 2012; Prins et al., 2008; Resende et al., 2011; Wickramasinghe et al., 2008). For instance, children from different populations vary in their limb dimensions and fat distribution, creating differences in impedance (Haroun et al., 2010). Kehoe et al. (2011) have shown that BIA, without a population specific calibration, is poor at predicting body fat percentage in Indian children. We have shown that the Tanita system’s built-in equations are inappropriate for children in Nepal, producing an average error of 0.3 kg, but at times as high as 4.5 kg, with the built-in equations tending to underestimate LM and potentially overestimate fat mass. To our knowledge, two other studies have produced prediction equations for LM from BIA in South Asian children: Khan et al. (2012) and Wickramasinghe et al. (2008). Both used the same parameters in their prediction equations. When applied to our cohort study they produced large errors (1.0 kg using the Bangladesh equation and 3.0 kg using the Sri Lanka equation). This emphasizes the need for population-specific equations, even for nearby populations.

If sufficient care is taken in the selection of participants and in carrying out the procedures, a prediction equation with a low standard error can be produced, giving greater confidence in body composition estimates. We made some improvements to the standard method to improve feasibility and accuracy. First, we removed weight error due to clothing by standardising it. Second, we recorded and standardised the intake of fluids. We did not measure urine output or water losses, but the study was done in winter with children indoors and unlikely to be sweating. Extreme hydration levels in the participants would have had an impact on the impedance values obtained. We had no reason to expect this, and level of hydration is not a requirement for using the equations. Third, we centrifuged saliva samples immediately to be able to secure a further sample if necessary. This resulted in no loss of data from inadequate volumes of saliva.

In addition, we examined the difference that arm position makes to LM estimates. The theory of segmental analysis divides the body into cylinders: the arms and legs, which are long and thin, with relatively high resistance, and the shorter, thicker trunk. Despite their low mass, the arms make up approximately 45% of whole body resistance (NIH Technology Assessment Statement, 1994). Small changes to arm position can introduce bias. Achieving a 90° angle accurately is difficult and we found that even small changes to wrist position affected the impedance values obtained by about 15 Ohms. To enable us to get BIA results from smaller children, we adapted the standard technique by flexing the arms to 90°. This reduced the impedance by 50–60 Ohms and extended the range of the Tanita BC-418 by approximately 5%, an important difference when dealing with undernourished or young children. The different arm position resulted in a small difference in LM overall, but when limiting this approach to children who weighed <16 kg, in whom this technique would be most useful, the methods appear to produce similar estimates. Specific isotope calibration is required if using a different arm position, or errors in the estimates can be large.

Because of the normal distribution of weight, sampling evenly across the weight range of a population adds substantially to the time and resources required to conduct an isotope calibration study. Finding adequate numbers of individuals in the tails of a weight distribution is difficult. To improve the feasibility of isotope calibration, we looked at the effects of reducing participant numbers. If time and resources permit, sampling across the weight range creates greater certainty in the regression line, highlighted by the narrow confidence intervals in our graphs. A normally distributed population sample would have included few smaller and larger children, making the regression line less stable. We showed that a reduction in numbers did result in greater error. The difference in the mean values was small, but the error became larger at the tails of the distribution. Limiting the number of individuals at the extremes of the data range could dramatically reduce time and resources needed to conduct the study but, as shown in the hypothetical scenarios created, does result in substantial error for heavier and lighter children. We would therefore recommend sampling across the entire weight range of the population.

The main strength of our study was the narrow age range chosen to calibrate the larger cohort, resulting in a low standard error. We cannot ascertain whether the prediction equations generated would be appropriate for different ages. While preferable to equations designed for other populations, there is limited generalizability to other age groups (Montagnese et al., 2013). It is also difficult to know whether the methodological aspects tested in our study would be applicable to more affluent populations with a wider range of FM and LM.

## Conclusions

We conducted an isotope calibration study to produce population-specific equations with low standard error for children in a larger cohort in Nepal. We believe that these are the first such equations produced for Nepalese children. We then showed how to improve BIA calibration studies by looking at posture and study design. Arm position is important, and while a smaller sample reduces resource requirements substantially, it results in large errors at the tails of the weight distribution.

## Supplemental Information

10.7717/peerj.785/supp-1 Text S1 Additional equations for the calculation of lean mass Equations for calculation of total body water and lean mass.

10.7717/peerj.785/supp-2 Table S1 Reduction of sample size: smaller range of weights Model fit and error in lean mass when collecting a smaller range of weights.

10.7717/peerj.785/supp-3 Table S2 Smaller sample size: reduced numbers at the tails of weight distribution Halving the number >1 sd from the mean.

10.7717/peerj.785/supp-4 Figure S1 Scatter plots for lean mass and impedance index Scatter plots, with regression lines, 95% confidence intervals and 95% prediction lines for lean mass from isotope dilution and impedance index (*Ht*^2^/*Z*).

10.7717/peerj.785/supp-5 Figure S2 Bland–Altman plots comparing standard and arm-position specific equations Comparison of lean mass results using standard (180°) calibration versus specific calibration equations.

10.7717/peerj.785/supp-6 Figure S3 Reduction of sample size: smaller range of weights Graphs showing impedance index against lean mass when smaller ranges of weights were tested.

10.7717/peerj.785/supp-7 Figure S4 Smaller sample size: reduced numbers at the tails of weight distribution Graphs showing impedance index against lean mass with reduced numbers in the tails of weight distribution.

10.7717/peerj.785/supp-8 Supplemental Information 8 Total body water and bioelectrical impedance data Supplemental file containing the total body water results and the bioelectrical impedance and anthropometry data.
