# Supplementary material for: Body composition in Nepalese children using isotope dilution: the production of ethnic-specific calibration equations and an exploration of methodological issues
Source: PeerJ. 2015 Mar 3;3:e785. doi: 10.7717/peerj.785 (PMC4358641; doi:10.7717/peerj.785)
Supplement: Text S1 — Equations for calculation of total body water and lean mass. [file peerj-03-785-s001.docx]

Text S1: Equations for calculation of total body water and lean mass

Total body water (N) was calculated using equation A:

(A)

$$N= \frac{TA}{a} \times\frac{(Ed-Et)}{(Es-Ep)}$$

where *T* is amount of tap water in which *a* is diluted before analysis, *A* is the mass of isotope drink given to the child in grams, *a* is the portion of the dose diluted in *T. E* values are isotope enrichments in delta (**δ)** units: *Ed* = average dose, *Ep* = average predose, *Es* = average post-dose, *Et* = average tap water

Lean mass was calculated using equation B:

(B)

$$\text{lean mass}= \frac{\left( \frac{\left( \frac{N}{1.044} \right)}{1000} \right)-0.23}{\text{age and sex specific hydration factors}}$$

where 1.044 is the correction factor to account for hydrogen ion exchange.[^1^](#_ENREF_1) With deuterium the dilution volume is larger than actual TBW volume because the labelled hydrogen atoms exchange with hydrogen atoms associated with carboxyl, hydroxyl, and amino groups. [^2^](#_ENREF_2) [^3^](#_ENREF_3)

1000 is needed to covert the figure into litres, 0.23 is the standardized volume of fluid drunk by the children in litres during the equilibration period. The age and sex specific hydration factors used were: 0.761 for boys and 0.753 for girls aged 6 to 7.99 and 0.758 for boys and 0.752 for girls aged 8 to 9.99 as described by Wells et al. [^4^](#_ENREF_4)

**References**

1. Racette SB, Schoeller DA, Luke AH, Shay K, Hnilicka J, Kushner RF. Relative dilution spaces of 2H- and 18O-labeled water in humans. *American Journal of Physiology - Endocrinology And Metabolism* 1994; **267**(4): E585-E90.

2. Culebras JM, Moore FD. Total body water and the exchangeable hydra- tion. I. Theoretical calculation of nonaqueous exchangeable hydration in man. . *Am J Physiol* 1977; **232**: 54-9.

3. Heymsfield SB, Matthews D. Body composition: research and clinical advances. . *J Parenter Enteral Nutr* 1994; **18**: 91–103.

4. Wells JC, Williams JE, Chomtho S, et al. Pediatric reference data for lean tissue properties: density and hydration from age 5 to 20 y. *Am J Clin Nutr* 2010; **91**(3): 610-8.
