# Supplementary material for: Body composition in Nepalese children using isotope dilution: the production of ethnic-specific calibration equations and an exploration of methodological issues
Source: PeerJ. 2015 Mar 3;3:e785. doi: 10.7717/peerj.785 (PMC4358641; doi:10.7717/peerj.785)
Supplement: Table S1 — Model fit and error in lean mass when collecting a smaller range of weights. [file peerj-03-785-s002.docx]

Supplemental Table S1: Model fit and error in lean mass when collecting a smaller range of weights

|  | Model 1  All data | Model 2  <1.5 SD | Model 3  <1.25 SD | Model 4  <1SD | Model 5  <0.75 SD |
| --- | --- | --- | --- | --- | --- |
| Number | 100 | 89 | 76 | 66 | 50 |
| ht^2^/Z | 0.681 | 0.641 | 0.639 | 0.592 | 0.618 |
| Weight | 0.211 | 0.254 | 0.264 | 0.309 | 0.326 |
| Sex | -0.363 | -0.233 | -0.311 | -0.255 | -0.297 |
| Constant | 1.946 | 1.550 | 1.455 | 1.221 | 0.375 |
| R^2^ | 0.93 | 0.94 | 0.92 | 0.89 | 0.84 |
| Root MSE | 0.95 | 0.79 | 0.78 | 0.81 | 0.87 |
| Residuals | 86.53 | 53.32 | 43.74 | 40.56 | 34.63 |
|  |  |  |  |  |  |
| Mean  (kg) | 17.32 | 17.21 | 17.24 | 17.17 | 17.09 |
| 95% confidence intervals | 17.13 to 17.51 | 17.01 to 17.41 | 17.04 to 17.44 | 16.97 to 17.37 | 16.88 to 17.30 |
| Standard deviation | 2.46 | 2.50 | 2.52 | 2.54 | 2.66 |
| Difference (g) from Model 1 |  | 113 | 82 | 149 | 231 |
| Percentage difference in mean |  | 0.7 | 0.5 | 0.9 | 1.3 |

Figure S3: Graphs showing impedance index against lean mass when smaller ranges of weights were tested
