# Supplementary material for: Body composition in Nepalese children using isotope dilution: the production of ethnic-specific calibration equations and an exploration of methodological issues
Source: PeerJ. 2015 Mar 3;3:e785. doi: 10.7717/peerj.785 (PMC4358641; doi:10.7717/peerj.785)
Supplement: Table S2 — Halving the number >1 sd from the mean. [file peerj-03-785-s003.docx]

Supplemental Table S2: Halving the number >1sd from the mean

|  | Model 1  All data | Model 2  half data >1SD | Model 3  half data >1SD | Model 4  half data >1SD | Model 5  half data >1SD |
| --- | --- | --- | --- | --- | --- |
| Number | 100 | 83 | 83 | 83 | 83 |
| ht^2^/Z | 0.681 | 0.636 | 0.731 | 0.656 | 0.681 |
| Weight | 0.211 | 0.250 | 0.150 | 0.220 | 0.229 |
| Sex | -0.363 | -0.196 | -0.397 | -0.503 | -0.341 |
| Constant | 1.946 | 1.691 | 2.416 | 2.235 | 1.514 |
| R^2^ | 0.93 | 0.93 | 0.91 | 0.93 | 0.93 |
| Root MSE | 0.95 | 0.85 | 0.93 | 0.87 | 0.93 |
| Residuals | 86.53 | 57.48 | 68.53 | 59.33 | 58.38 |
|  |  |  |  |  |  |
| Mean  (kg) | 17.32 | 17.21 | 17.39 | 17.33 | 17.28 |
| 95% confidence intervals | 17.13 to 17.51 | 17.02 to 17.40 | 17.20 to 17.58 | 17.14 to 17.52 | 17.08 to 17.48 |
| Standard deviation | 2.46 | 2.48 | 2.41 | 2.41 | 2.52 |
| Difference (g) from Model 1 |  | 112 | 66 | 10 | 40 |
| Percentage difference in mean |  | 0.7 | 0.4 | 0.1 | 0.2 |
