# Supplementary material for: Body composition in Nepalese children using isotope dilution: the production of ethnic-specific calibration equations and an exploration of methodological issues
Source: PeerJ. 2015 Mar 3;3:e785. doi: 10.7717/peerj.785 (PMC4358641; doi:10.7717/peerj.785)
Supplement: Supplemental Information 8 — Supplemental file containing the total body water results and the bioelectrical impedance and anthropometry data. [file peerj-03-785-s008.pdf]

Supplemental file: Total body water laboratory data

Sample Details

Number: 306
Material: Saliva and doses

Analysis Details

Isotope(s) : Deuterium
Method: Equilibration IRMS
Report Date: 4-May-2012

Table 1: Results of deuterium analysis of TBW samples

Subject ID	Dose weight (g/100 ml)	Tap Water used	Time Point	Deuterium Result δ^2H vs V-SMOW (‰)	^2H (ppm)
CL 001	0.2018	1	Dose	108.98	172.71
				109.20	172.74
			Pre	-25.80	151.72
				-26.52	151.61
			Post	498.82	233.40
				496.03	232.97
CL 002	0.2303	1	Dose	123.43	174.95
				123.77	175.01
			Pre	-23.51	152.07
				-24.82	151.87
			Post	486.42	231.47
				488.58	231.81
CL 003	0.2013	1	Dose	92.18	170.09
				95.22	170.56
			Pre	-23.26	152.11
				-21.21	152.43
			Post	506.54	234.60
				507.92	234.82
CL 004	0.202	1	Dose	111.18	173.05
				109.89	172.85
			Pre	-17.96	152.94
				-18.44	152.86
			Post	597.41	248.75

				599.90	249.14
CL 005	0.2012	1	Dose	98.24	171.03
				99.87	171.29
			Pre	-21.52	152.38
				-25.10	151.83
			Post	563.83	243.52
				566.12	243.88
CL 006	0.2012	1	Dose	104.87	172.07
				106.68	172.35
			Pre	-15.19	153.37
				-15.44	153.33
			Post	558.56	242.70
				556.91	242.45
CL 007	0.2014	1	Dose	114.68	173.59
				112.86	173.31
			Pre	-24.03	151.99
				-24.39	151.94
			Post	489.21	231.91
				488.27	231.76
CL 008	0.2017	1	Dose	111.01	173.02
				110.51	172.94
			Pre	-26.08	151.67
				-28.08	151.36
			Post	473.70	229.49
				473.96	229.53
CL 009	0.2013	1	Dose	99.70	171.26
				100.84	171.44
			Pre	-32.06	150.74
				-31.87	150.77
			Post	522.03	237.02
				518.93	236.53
CL 010	0.2017	1	Dose	101.50	171.54
				97.31	170.89
			Pre	-24.40	151.94
				-26.31	151.64
			Post	511.36	235.35
				511.19	235.33

CL 011	0.2009	1	Dose	95.72	170.64
				94.44	170.44
			Pre	-18.40	152.87
				-19.85	152.65
			Post	455.81	226.71
				454.64	226.52
CL 012	0.2008	1	Dose	100.46	171.38
				99.86	171.28
			Pre	-21.40	152.40
				-22.13	152.29
			Post	489.91	232.01
				489.46	231.94
CL 013	0.2016	1	Dose	94.89	170.51
				94.44	170.44
			Pre	-23.19	152.12
				-22.69	152.20
			Post	530.65	238.36
				532.00	238.57
CL 014	0.2009	1	Dose	94.94	170.52
				94.00	170.37
			Pre	-29.15	151.20
				-28.00	151.38
			Post	625.17	253.07
				626.42	253.27
CL 015	0.2013	1	Dose	118.36	174.17
				118.20	174.14
			Pre	-29.30	151.17
				-29.14	151.20
			Post	474.78	229.66
				476.10	229.86
CL 016	0.2018	1	Dose	101.86	171.60
				102.93	171.76
			Pre	-25.42	151.78
				-26.24	151.65
			Post	553.91	241.98
				555.91	242.29
CL 017	0.2002	1	Dose	110.51	172.94

				109.22	172.74
			Pre	-23.49	152.08
				-24.47	151.93
			Post	739.56	270.88
				744.22	271.61
CL 018	0.201	1	Dose	105.21	172.12
				103.85	171.91
			Pre	-26.92	151.54
				-24.77	151.88
			Post	554.70	242.10
				555.11	242.17
CL 020	0.2025	1	Dose	100.35	171.36
				100.61	171.40
			Pre	-27.90	151.39
				-23.02	152.15
			Post	449.47	225.72
				449.63	225.74
CL 021	0.2014	1	Dose	100.67	171.41
				100.66	171.41
			Pre	-22.51	152.23
				-23.20	152.12
			Post	510.50	235.22
				507.21	234.71
CL 022	0.2011	1	Dose	93.43	170.28
				89.81	169.72
			Pre	-21.22	152.43
				-21.79	152.34
			Post	624.81	253.02
				626.46	253.27
CL 023	0.2012	1	Dose	113.46	173.40
				110.45	172.93
			Pre	-20.72	152.51
				-20.36	152.56
			Post	550.19	241.40
				548.45	241.13
CL 024	0.2016	2	Dose	97.65	170.94
				96.20	170.71

			Pre	-23.57	152.07
				-23.84	152.02
			Post	536.65	239.29
				539.43	239.72
CL 025	0.1994	2	Dose	110.90	173.00
				109.39	172.77
			Pre	-21.91	152.32
				-22.97	152.16
			Post	469.45	228.83
				471.62	229.17
CL 026	0.202	2	Dose	102.09	171.63
				98.42	171.06
			Pre	-21.49	152.39
				-21.60	152.37
			Post	546.35	240.80
				552.03	241.69
CL 027	0.2033	2	Dose	99.82	171.28
				100.76	171.43
			Pre	-17.39	153.03
				-15.94	153.25
			Post	571.78	244.76
				568.15	244.20
CL 028	0.2021	2	Dose	107.18	172.43
				105.92	172.23
			Pre	-22.04	152.30
				-24.32	151.95
			Post	528.87	238.08
				530.67	238.36
CL 029	0.2032	2	Dose	119.38	174.32
				119.39	174.33
			Pre	-20.90	152.48
				-24.21	151.97
			Post	384.85	215.66
				383.57	215.46
CL 030	0.2029	2	Dose	110.34	172.92
				109.62	172.80
			Pre	-24.13	151.98

				-24.35	151.94
			Post	574.86	245.24
				573.42	245.02
CL 032	0.203	2	Dose	116.21	173.83
				115.31	173.69
			Pre	-27.64	151.43
				-28.50	151.30
			Post	496.47	233.04
				497.84	233.25
CL 033	0.2034	2	Dose	103.85	171.91
				103.37	171.83
			Pre	-24.82	151.87
				-24.78	151.88
			Post	478.81	230.29
				477.34	230.06
CL 034	0.2034	2	Dose	113.98	173.48
				112.38	173.23
			Pre	-27.73	151.42
				-28.87	151.24
			Post	676.21	261.02
				678.74	261.41
CL 035	0.2024	2	Dose	104.44	172.00
				106.60	172.33
			Pre	-15.95	153.25
				-19.42	152.71
			Post	705.06	265.51
				706.08	265.67
CL 036	0.2018	2	Dose	93.75	170.33
				97.52	170.92
			Pre	-26.53	151.60
				-24.03	151.99
			Post	609.41	250.62
				611.19	250.90
CL 037	0.2023	2	Dose	114.13	173.51
				112.25	173.21
			Pre	-20.46	152.55
				-19.78	152.66

			Post	496.02	232.97
				496.27	233.00
CL 039	0.2041	5	Dose	109.41	172.77
		(analysed out of sequence		107.92	172.54
		hence different tap no.)	Pre	-24.42	151.93
				-26.47	151.61
			Post	542.58	240.21
				544.28	240.48
CL 040	0.2028	2	Dose	97.43	170.91
				98.69	171.10
			Pre	-17.39	153.03
				-14.69	153.45
			Post	452.16	226.14
				453.47	226.34
CL 041	0.2026	2	Dose	109.55	172.79
				108.22	172.59
			Pre	-22.64	152.21
				-26.00	151.69
			Post	528.84	238.08
				529.45	238.17
CL 042	0.2028	2	Dose	110.78	172.98
				111.56	173.11
			Pre	-28.35	151.32
				-30.16	151.04
			Post	682.98	262.07
				688.22	262.89
CL 043	0.2029	2	Dose	110.69	172.97
				109.27	172.75
			Pre	-18.64	152.83
				-20.12	152.60
			Post	434.85	223.44
				433.34	223.21
CL 044	0.2049	2	Dose	98.01	171.00
				98.77	171.12
			Pre	-24.64	151.90
				-24.57	151.91
			Post	489.13	231.89

				488.49	231.79
CL 045	0.2045	2	Dose	108.79	172.67
				106.02	172.24
			Pre	-19.84	152.65
				-20.59	152.53
			Post	426.16	222.09
				426.11	222.08
CL 046	0.2021	2	Dose	92.83	170.19
				93.20	170.25
			Pre	-22.48	152.24
				-25.45	151.77
			Post	570.95	244.63
				571.55	244.72
CL 047	0.2022	2	Dose	100.46	171.38
				101.75	171.58
			Pre	-26.74	151.57
				-27.53	151.45
			Post	442.75	224.67
				441.96	224.55
CL 048	0.2026	2	Dose	101.14	171.48
				99.50	171.23
			Pre	-28.43	151.31
				-27.54	151.45
			Post	441.37	224.46
				441.49	224.48
CL 049	0.2021	3	Dose	101.05	171.47
				97.96	170.99
			Pre	-24.27	151.96
				-25.12	151.82
			Post	435.36	223.52
				436.22	223.66
CL 050	0.2038	3	Dose	95.30	170.57
				95.32	170.58
			Pre	-16.55	153.16
				-18.65	152.83
			Post	631.82	254.11
				630.79	253.95

CL 051	0.2045	3	Dose	113.01	173.33
				110.97	173.01
			Pre	-22.58	152.22
				-21.12	152.45
			Post	629.89	253.81
				628.80	253.64
CL 052	0.2023	3	Dose	98.62	171.09
				96.92	170.83
			Pre	-23.49	152.08
				-24.90	151.86
			Post	623.35	252.79
				623.25	252.77
CL 053	0.2025	3	Dose	124.75	175.16
				122.85	174.86
			Pre	-26.00	151.69
				-28.20	151.34
			Post	581.61	246.29
				583.91	246.65
CL 054	0.203	3	Dose	125.29	175.24
				128.33	175.72
			Pre	-26.01	151.69
				-28.51	151.30
			Post	429.67	222.64
				430.43	222.75
CL 055	0.2042	3	Dose	124.48	175.12
				125.92	175.34
			Pre	-17.18	153.06
				-19.46	152.71
			Post	585.74	246.93
				589.69	247.55
CL 056	0.2044	3	Dose	123.55	174.97
				123.76	175.01
			Pre	-26.86	151.55
				-30.01	151.06
			Post	335.27	207.94
				335.33	207.95
CL 057	0.2028	3	Dose	108.90	172.69

				107.14	172.42
			Pre	-25.20	151.81
				-24.49	151.92
			Post	453.57	226.36
				455.74	226.69
CL 058	0.2042	3	Dose	116.38	173.86
				115.60	173.74
			Pre	-27.44	151.46
				-30.24	151.03
			Post	327.50	206.73
				328.90	206.95
CL 059	0.2027	3	Dose	114.73	173.60
				114.41	173.55
			Pre	-29.93	151.07
				-30.62	150.97
			Post	357.18	211.35
				357.79	211.44
CL 060	0.2044	3	Dose	113.89	173.47
				112.92	173.32
			Pre	-24.09	151.98
				-23.77	152.03
			Post	380.12	214.92
				384.30	215.57
CL 061	0.2043	3	Dose	121.66	174.68
				120.90	174.56
			Pre	-24.50	151.92
				-25.96	151.69
			Post	420.87	221.27
				421.83	221.42
CL 062	0.2034	3	Dose	110.82	172.99
				112.30	173.22
			Pre	-24.04	151.99
				-26.36	151.63
			Post	347.86	209.90
				349.15	210.10
CL 063	0.2018	3	Dose	110.76	172.98
				109.96	172.86

			Pre	-25.52	151.76
				-26.42	151.62
			Post	456.90	226.87
				459.12	227.22
CL 064	0.2015	3	Dose	92.33	170.11
				90.77	169.87
			Pre	-25.01	151.84
				-23.35	152.10
			Post	661.89	258.79
				663.70	259.07
CL 065	0.2019	3	Dose	114.24	173.52
				114.50	173.56
			Pre	-9.19	154.30
				-13.18	153.68
			Post	476.65	229.95
				476.76	229.97
CL 066	0.2018	3	Dose	101.48	171.54
				101.94	171.61
			Pre	-23.65	152.05
				-25.82	151.72
			Post	552.66	241.78
				551.58	241.62
CL 067	0.2019	3	Dose	104.12	171.95
				105.64	172.18
			Pre	-25.18	151.82
				-26.88	151.55
			Post	395.81	217.36
				396.45	217.46
CL 068	0.2023	3	Dose	120.44	174.49
				120.66	174.52
			Pre	-28.44	151.31
				-28.36	151.32
			Post	506.56	234.61
				507.96	234.82
CL 069	0.2014	3	Dose	97.21	170.87
				97.97	170.99
			Pre	-24.28	151.96

				-23.66	152.05
			Post	459.54	227.29
				461.01	227.51
CL 070	0.2012	3	Dose	108.86	172.69
				109.30	172.75
			Pre	-18.10	152.92
				-21.95	152.32
			Post	445.47	225.10
				446.78	225.30
CL 071	0.2001	4	Dose	126.39	175.42
				124.52	175.12
			Pre	-20.30	152.58
				-19.91	152.64
			Post	750.15	272.53
				750.08	272.52
CL 073	0.2012	4	Dose	112.04	173.18
				113.84	173.46
			Pre	-16.63	153.15
				-19.18	152.75
			Post	363.41	212.32
				363.70	212.37
CL 074	0.201	4	Dose	97.89	170.98
				97.95	170.99
			Pre	-19.76	152.66
				-20.42	152.56
			Post	396.78	217.51
				395.25	217.28
CL 075	0.2012	4	Dose	99.36	171.21
				99.82	171.28
			Pre	-24.72	151.89
				-22.69	152.20
			Post	353.00	210.70
				354.01	210.86
CL 076	0.2015	4	Dose	106.57	172.33
				108.23	172.59
			Pre	-25.99	151.69
				-30.53	150.98

			Post	378.30	214.64
				377.20	214.47
CL 077	0.2013	4	Dose	116.84	173.93
				114.74	173.60
			Pre	-19.25	152.74
				-22.40	152.25
			Post	362.25	212.14
				364.32	212.46
CL 078	0.2018	4	Dose	102.22	171.65
				100.63	171.40
			Pre	-24.92	151.86
				-27.49	151.46
			Post	351.36	210.44
				354.51	210.93
CL 079	0.2007	4	Dose	107.66	172.50
				106.32	172.29
			Pre	-22.14	152.29
				-23.56	152.07
			Post	491.54	232.27
				491.01	232.19
CL 080	0.2016	4	Dose	90.34	169.80
				90.06	169.76
			Pre	-33.08	150.59
				-35.74	150.17
			Post	401.38	218.23
				398.80	217.83
CL 081	0.201	4	Dose	98.67	171.10
				98.14	171.02
			Pre	-20.92	152.48
				-19.50	152.70
			Post	434.13	223.33
				436.55	223.71
CL 083	0.2011	4	Dose	103.43	171.84
				102.42	171.68
			Pre	-21.09	152.45
				-24.24	151.96
			Post	406.82	219.08

				405.11	218.81
CL 084	0.2016	4	Dose	119.42	174.33
				118.20	174.14
			Pre	-18.67	152.83
				-22.34	152.26
			Post	372.18	213.69
				373.11	213.83
CL 085	0.2012	4	Dose	98.07	171.01
				96.69	170.79
			Pre	-27.11	151.51
				-27.64	151.43
			Post	423.57	221.69
				423.45	221.67
CL 087	0.2008	4	Dose	103.76	171.89
				104.63	172.03
			Pre	-23.54	152.07
				-23.16	152.13
			Post	488.43	231.78
				488.89	231.86
CL 089	0.2015	4	Dose	110.19	172.89
				107.61	172.49
			Pre	-18.31	152.88
				-16.97	153.09
			Post	488.24	231.75
				486.92	231.55
CL 090	0.2018	4	Dose	108.99	172.71
				109.80	172.83
			Pre	-22.15	152.29
				-25.58	151.75
			Post	615.32	251.54
				621.83	252.55
CL 091	0.2011	4	Dose	100.79	171.43
				98.48	171.07
			Pre	-14.20	153.52
				-17.38	153.03
			Post	428.97	222.53
				431.13	222.86

CL 092	0.2009	4	Dose	99.57	171.24
				98.72	171.11
			Pre	-22.78	152.19
				-24.40	151.94
			Post	391.33	216.67
				394.31	217.13
CL 093	0.2012	4	Dose	93.68	170.32
				95.33	170.58
			Pre	-27.88	151.39
				-28.60	151.28
			Post	663.69	259.07
				668.58	259.83
CL 094	0.2007	4	Dose	95.95	170.68
				99.71	171.26
			Pre	-22.92	152.17
				-24.68	151.89
			Post	658.03	258.19
				654.39	257.62
CL 095	0.2014	4	Dose	97.66	170.94
				101.11	171.48
			Pre	-26.35	151.63
				-28.13	151.36
			Post	377.04	214.44
				373.38	213.87
CL 096	0.2013	4	Dose	94.32	170.42
				94.65	170.47
			Pre	-19.84	152.65
				-22.60	152.22
			Post	616.72	251.76
				614.00	251.33
CL 097	0.2012	5	Dose	89.83	169.72
				90.14	169.77
			Pre	-18.18	152.90
				-19.06	152.77
			Post	389.00	216.30
				391.43	216.68
CL 098	0.2022	5	Dose	108.84	172.68

				107.49	172.47
			Pre	-26.64	151.59
				-27.66	151.43
			Post	370.76	213.46
				371.41	213.56
CL 099	0.2054	5	Dose	108.92	172.70
				109.17	172.73
			Pre	-24.29	151.95
				-25.04	151.84
			Post	445.60	225.12
				445.05	225.03
CL 100	0.2036	5	Dose	94.22	170.41
				93.86	170.35
			Pre	-27.45	151.46
				-30.79	150.94
			Post	326.29	206.54
				331.24	207.31
CL 101	0.2035	5	Dose	93.19	170.25
				94.74	170.49
			Pre	-18.61	152.84
				-20.80	152.50
			Post	412.78	220.01
				414.08	220.21
CL 102	0.2041	5	Dose	99.31	171.20
				99.53	171.23
			Pre	-12.88	153.73
				-13.18	153.68
			Post	677.99	261.30
				681.43	261.83
CL 103	0.2065	5	Dose	106.82	172.37
				103.80	171.90
			Pre	-22.13	152.29
				-22.15	152.29
			Post	621.94	252.57
				622.20	252.61
CL 104	0.207	5	Dose	103.80	171.90
				104.52	172.01

			Pre	-18.90	152.79
				-22.84	152.18
			Post	637.05	254.92
				638.06	255.08
CL 105	0.2036	5	Dose	97.17	170.87
				95.54	170.61
			Pre	-23.13	152.13
				-24.81	151.87
			Post	417.24	220.70
				415.82	220.48
CL 106	0.2047	5	Dose	108.30	172.60
				109.39	172.77
			Pre	-27.45	151.46
				-26.18	151.66
			Post	369.99	213.34
				369.45	213.26
CL 107	0.2046	5	Dose	121.22	174.61
				119.45	174.34
			Pre	-16.61	153.15
				-17.24	153.05
			Post	791.99	279.04
				787.20	278.30
CL 108	0.2023	5	Dose	100.54	171.39
				100.38	171.37
			Pre	-19.69	152.67
				-19.39	152.72
			Post	681.31	261.81
				680.49	261.68
CL 109	0.2025	5	Dose	90.04	169.76
				90.09	169.76
			Pre	-22.18	152.28
				-23.52	152.07
			Post	701.83	265.01
				700.78	264.84

Tap Waters for Dose Dilutions

Tap Water Number				Deuterium Result<br>δ²H vs V-SMOW (‰)	 ²H (ppm)
1				-35.53	150.20
				-38.09	149.80
2				-37.98	149.82
				-38.30	149.77
3				-39.15	149.64
				-39.69	149.56
4				-39.28	149.62
				-41.13	149.33
5				-41.36	149.30
				-41.26	149.31

Results for control samples

Batch Number				Deuterium Result<br>δ²H vs V-SMOW (‰)	 ²H (ppm)
1				840.29	286.56
				841.64	286.77
				843.78	287.10
				841.60	286.77
2				838.95	286.35
				843.14	287.00
				845.09	287.31
				846.43	287.52
				843.46	287.06
				845.23	287.33
				842.51	286.91
				844.30	287.18
3				847.37	287.66
				847.95	287.75
				846.74	287.56
				847.88	287.74
4				844.79	287.26
				843.19	287.01
				842.94	286.97
				848.03	287.77

		838.68	286.31
		843.81	287.11
		844.03	287.14
		844.86	287.27
5		838.56	286.29
		842.82	286.95
		840.51	286.60
		843.74	287.10
		845.50	287.37
		845.29	287.34
		844.06	287.15
		847.16	287.63
6		848.59	287.85
		848.39	287.82
		848.25	287.80
		847.73	287.72
		842.48	286.90
		844.01	287.14
	Mean	844.31	287.19
	SD	2.79	0.43
	n	38	38
	Calibrated value	843.43	287.05

Supplemental file: Bioelectrical impedance data

ID number	DOB	Sex (M=1,F=0)	Date	Weight (kg)	Standing height (cm)
1	05/08/2003	0	29/01/12	22.6	125.8
2	16/05/2003	0	29/01/12	21.9	126.3
3	14/01/2004	1	29/01/12	20.1	117.9
4	29/01/2003	1	29/01/12	18.5	112.7
5	29/01/2003	1	29/01/12	18.7	115.6
6	06/02/2004	1	29/01/12	18.0	112.2
7	06/02/2003	0	29/01/12	21.8	122.5
8	09/04/2003	1	29/01/12	21.1	122.7
9	28/06/2003	0	29/01/12	18.0	113.7
10	09/03/2004	0	29/01/12	20.9	117.8
11	25/04/2003	1	29/01/12	22.3	127.6
12	03/09/2003	1	29/01/12	19.9	121.3
13	17/11/2003	0	29/01/12	18.7	117.6
14	30/06/2003	0	29/01/12	16.1	116.2
15	11/11/2003	1	29/01/12	20.6	121.0
16	16/09/2003	1	29/01/12	20.3	118.0
17	27/04/2004	1	29/01/12	18.5	118.4
18	05/02/2003	0	29/01/12	14.3	109.9
19	29/10/2003	1	29/01/12	22.3	126.3
20	24/03/2003	1	29/01/12	21.8	123.4
21	26/06/2003	1	29/01/12	19.9	121.0
22	27/06/2003	0	29/01/12	16.9	117.0
23	14/11/2003	0	29/01/12	19.7	120.0
24	19/09/2003	0	29/01/12	18.9	121.9
25	12/10/2003	1	29/01/12	21.7	125.7
26	17/05/2003	0	29/01/12	20.0	115.7
27	23/01/2003	0	29/01/12	19.5	118.4
28	15/02/2003	0	29/01/12	19.4	112.9
29	26/02/2003	0	29/01/12	25.6	128.3
30	17/03/2004	1	29/01/12	17.7	116.4

31	01/03/2004	1	29/01/12	19.4	108.4
32	20/06/2003	0	29/01/12	23.4	126.4
33	28/01/2003	0	29/01/12	20.9	125.4
34	13/04/2003	1	29/01/12	17.7	112.6
35	27/10/2003	0	29/01/12	16.1	111.8
36	17/08/2003	0	29/01/12	19.3	114.8
37	22/02/2003	0	29/01/12	21.7	124.7
38	04/07/2003	1	29/01/12	22.1	124.0
39	01/11/2003	0	29/01/12	17.7	119.4
40	14/02/2003	1	29/01/12	22.5	125.3
41	21/01/2003	1	29/01/12	19.3	116.3
42	09/02/2003	0	30/01/12	18.2	115.9
43	16/07/2003	0	30/01/12	26.2	124.2
44	17/04/2003	1	30/01/12	20.2	122.0
45	27/06/2003	0	30/01/12	25.6	131.9
46	11/03/2004	1	30/01/12	17.6	115.6
47	19/02/2003	1	30/01/12	22.0	123.0
48	22/02/2003	0	30/01/12	25.3	126.8
49	07/08/2003	1	30/01/12	23.0	124.4
50	28/05/2003	0	30/01/12	17.7	116.3
51	15/04/2003	0	30/01/12	16.4	115.0
52	12/04/2003	0	30/01/12	15.9	118.4
53	27/11/2004	0	30/01/12	17.8	112.0
54	05/12/2003	1	30/01/12	24.0	128.5
55	01/08/2003	1	30/01/12	17.9	122.3
56	25/07/2003	1	30/01/12	31.9	134.5
57	8 year	1	31/01/12	31.7	126.4
58	09/06/2003	1	31/01/12	34.5	131.6
59	24/03/2004	1	31/01/12	26.4	128.4
60	9 years	1	31/01/12	25.5	130.6
61	22/09/2004	1	31/01/12	24.9	122.5
62	04/03/2003	1	31/01/12	29.1	135.6

63	09/02/2002	0	31/01/12	24.5	131.0
64	01/09/2003	0	31/01/12	14.3	103.7
65	16/12/2002	0	01/02/12	23.4	119.9
66	16/01/2004	0	01/02/12	17.4	115.8
67	9 years	1	02/02/12	31.1	127.4
68	02/07/2003	0	03/02/12	22.2	123.2
69	24/11/2003	0	03/02/12	24.4	124.3
70	9 years	0	03/02/12	25.1	129.2
71	28/10/2003	1	03/02/12	15.1	107.8
72	8 years	1	04/02/12	24.1	133.2
73	8 years	1	04/02/12	33.4	133.9
74	9 years	1	04/02/12	26.1	136.0
75	9 years	1	04/02/12	30.3	141.1
76	9 years	1	04/02/12	27.1	138.1
77	9 years	0	04/02/12	28.6	141.2
78	9 years	0	04/02/12	29.8	141.3
79	9 years	0	04/02/12	22.1	130.4
80	9 years	0	04/02/12	26.0	137.9
81	8 years	1	04/02/12	24.2	124.2
82	9 years	0	04/02/12	22.0	129.2
83	07/04/2003	1	05/02/12	26.7	124.1
84	9 years	1	06/02/12	29.7	140.7
85	9 years	0	06/02/12	23.2	128.7
86	Removed from study as too old. Did not receive isotope.		06/02/12		
87	9 years	1	06/02/12	22.9	128.1
88	9 years	1	06/02/12	24.9	129.7
89	9 years	1	06/02/12	25.4	119.4
90	7 years	1	06/02/12	15.9	111.9
91	9 years	1	06/02/12	22.6	131.4
92	9 years	1	06/02/12	28.4	139.7
93	years	1	06/02/12	15.8	116.4
94	7 years	1	06/02/12	16.0	104.0

95	29/03/2003	1	06/02/12	29.1	131.0
96	28/11/03	0	07/02/12	16.4	113.2
97	05/11/2003	0	08/02/12	26.7	134.2
98	9 years	1	08/02/12	34.3	142.3
99	14/07/2003	0	08/02/12	27.5	127.2
100	9 years	1	08/02/12	28.8	134.8
101	9 years	0	08/02/12	29.2	130.0
102	7 years	1	08/02/12	15.4	103.9
103	7 years	0	09/02/12	15.3	111.4
104	7 years	0	09/02/12	15.5	106.8
105	9 years	0	10/02/12	28.3	134.2
106	27/08/2003	0	10/02/12	31.9	129.3
107	7 years	0	10/02/12	14.2	112.7
108	7 years	0	10/02/12	15.6	106.5
109	7 years	0	10/02/12	14.4	107.8

ID number	Arms 90 degrees Whole body impedance (Ohms)	Right leg imp (Ohms)	Left leg imp (Ohms)	Right arm imp (Ohms)	Left arm imp (Ohms)
1	935	402	397	493	506
2	923	361	375	516	514
3	828	330	335	484	476
4	906	403	398	481	474
5	919	418	409	483	485
6	798	346	342	448	429
7	829	339	331	486	468
8	876	358	358	507	486
9	888	377	387	440	479
10	844	368	364	461	449
11	879	386	397	460	457
12	887	365	367	483	496
13	888	391	392	459	465
14	1128	483	479	621	603
15	940	390	392	493	527
16	873	364	363	465	486
17	904	394	386	475	487
18	1112	488	501	575	577
19	832	373	369	449	437
20	792	330	319	436	447
21	875	402	395	465	458
22	1089	473	472	581	592
23	943	421	417	494	502
24	1038	427	449	573	559
25	936	404	404	523	509
26	975	427	429	530	517
27	898	398	391	480	478
28	846	375	380	432	446
29	742	309	306	431	414
30	996	464	463	533	499

31	Unable to do due to developmental delay. Did not have the isotope.				
32	891	348	352	528	518
33	908	371	389	488	492
34	875	392	391	449	455
35	1094	483	476	581	589
36	1036	450	444	541	556
37	925	410	405	493	492
38	836	359	357	461	448
39	957	393	394	546	531
40	861	354	363	481	471
41	800	351	348	430	428
42	1090	508	510	566	543
43	786	318	309	457	460
44	857	354	354	464	476
45	870	360	370	461	474
46	885	396	393	483	463
47	784	333	335	419	418
48	811	333	344	460	457
49	744	316	310	419	405
50	1024	438	461	575	548
51	959	412	408	563	525
52	1148	492	478	626	623
53	827.0	366.0	363.0	487.0	445.0
54	774	326	327	423	423
55	1146	475	473	634	643
56	814	345	350	437	453
57	779	295	292	456	454
58	700	296	294	400	386
59	677	284	282	388	373
60	764	336	340	405	405
61	754	321	318	392	406
62	776	344	344	417	409

63	970	379	382	560	557
64	993	414	398	554	553
65	823	341	352	426	445
66	964	385	392	534	541
67	757	292	302	416	428
68	960	415	431	518	504
69	856	354	352	474	473
70	892	389	395	455	474
71	828	352	353	428	445
72	975	369	370	548	572
73	752	307	306	413	420
74	835	346	354	447	456
75	898	384	382	488	490
76	816	341	339	459	449
77	845	363	361	451	456
78	858	334	353	461	479
79	982	422	417	528	531
80	895	363	369	496	494
81	734	318	320	407	407
82	911	382	388	496	498
83	839	359	354	453	456
84	788	307	302	449	453
85	883	369	369	478	485
86					
87	883	350	341	497	510
88	767	321	328	422	405
89	738	291	295	417	411
90	928	384	375	522	527
91	855	376	376	468	447
92	879	353	353	478	493
93	1106	471	461	619	610
94	868	360	358	504	484

95	785	324	329	424	442
96	992	404	394	566	565
97	852	360	359	456	463
98	831	351	342	449	462
99	821	348	345	442	450
100	728	314	308	398	398
101	801	347	358	411	417
102	957	424	418	567	516
103	997	422	443	524	528
104	874	377	389	465	465
105	871	388	389	490	453
106	791	336	343	449	426
107	1114	468	459	596	617
108	995	449	458	508	497
109	1173	485	517	618	625

ID number	Arms 180 degrees Whole body impedance (Ohms)	Right leg imp (Ohms)	Left leg imp (Ohms)	Right arm imp (Ohms)	Left arm imp (Ohms)
1	1000	402	397	544	583
2	1011	363	377	576	603
3	915	329	336	541	571
4	968	408	397	553	542
5	992	417	410	582	560
6	882	344	342	528	520
7	896	340	334	526	530
8	947	356	361	566	548
9	953	373	384	524	551
10	909	373	366	509	511
11	955	390	398	517	522
12	951	364	366	550	562
13	968	392	395	548	552
14	1194	483	479	685	683
15	979	393	391	541	556
16	952	363	361	535	568
17	965	394	387	550	557
18	1178	484	499	627	648
19	879	370	369	467	502
20	850	330	318	486	515
21	942	395	389	541	545
22	1166	473	475	660	644
23	1003	417	413	553	572
24	1121	431	450	652	651
25	990	404	403	565	561
26	1040	429	430	590	577
27	950	395	390	540	538
28	906	376	386	504	496
29	818	307	302	498	486
30	1056	462	464	585	556

31						
32		985	345	351	593	613
33		995	370	391	577	592
34		943	391	391	517	535
35		1148	474	468	630	647
36		1103	450	448	589	623
37		984	414	402	558	555
38		901	364	358	515	515
39		990	390	392	585	573
40		949	357	366	553	555
41		865	351	348	498	506
42		1158	511	511	606	610
43		846	317	306	501	476
44		946	353	353	538	565
45		926	361	370	521	527
46		971	393	390	562	558
47		832	336	334	458	494
48		892	333	344	521	544
49		794	315	310	475	464
50		1134	444	462	634	664
51		1065	415	410	620	629
52	No result					
53		860	367	365	469	478
54		805	325	327	486	459
55		1182	472	471	694	683
56		888	346	350	504	529
57		859	297	295	510	537
58		755	296	293	470	440
59		715	284	283	431	417
60		812	334	340	458	463
61		805	324	320	445	458
62		831	346	345	462	468

63	1049	382	380	611	641
64	1042	418	401	605	607
65	890	341	353	475	512
66	1036	383	394	606	617
67	794	290	302	448	460
68	1023	411	425	559	567
69	905	354	353	523	520
70	945	389	394	506	524
71	883	347	353	481	502
72	1031	368	368	616	659
73	795	307	303	452	474
74	887	343	352	506	512
75	951	381	381	547	538
76	889	342	339	513	524
77	913	361	361	507	527
78	909	333	349	524	533
79	1067	420	418	593	613
80	970	361	368	552	590
81	794	318	322	461	447
82	986	384	389	564	586
83	862	361	354	475	480
84	827	307	305	489	490
85	944	371	375	534	548
86					
87	921	351	342	539	550
88	814	321	329	476	459
89	779	290	295	463	456
90	962	381	375	583	572
91	912	381	382	509	501
92	911	349	350	525	524
93	1186	472	464	694	694
94	965	361	350	568	580

95		828	320	326	466	468
96		1036	400	395	614	615
97		873	361	360	507	514
98		890	348	339	486	520
99		868	349	343	481	504
100		783	313	308	455	451
101		843	344	356	451	457
102		1048	428	419	606	618
103		1058	419	437	553	607
104		912	377	390	515	501
105		921	392	391	538	500
106		859	338	350	487	486
107		1174	468	460	670	680
108		1042	454	463	588	574
109	No result					
